# Open questions: Chromosome condensation - Why does a chromosome look like a chromosome?

**DOI:** 10.1186/1741-7007-11-9

**Published:** 2013-01-31

**Authors:** Frank Uhlmann

**Affiliations:** 1 Chromosome Segregation Laboratory, Cancer Research UK London Research Institute, 44 Lincoln's Inn Fields, London WC2A 3LY, UK

## 

**Figure 1:**
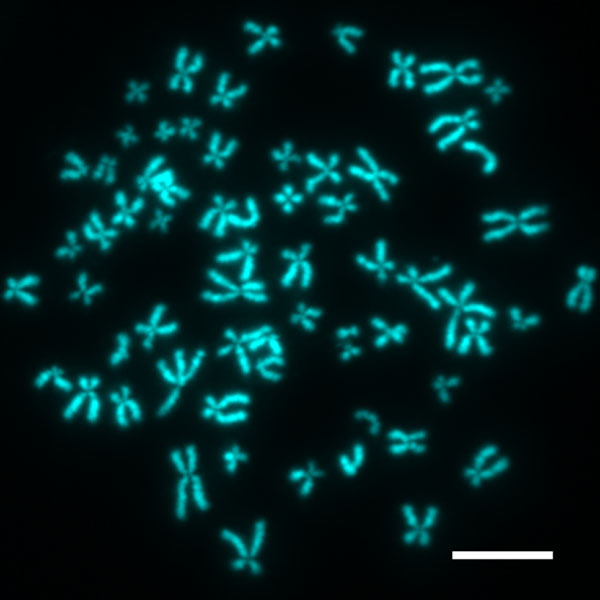
**Human chromosomes**. Meters of DNA are packed into micrometer-sized chromosomes in mitosis, here in human (HeLa) cells arrested in mitosis. Chromosomes were spread and stained with a DNA binding dye. Scale bar, 10 μm (photograph courtesy of Sriramkumar Sundaramoorthy and Mark Petronczki).

The x-shaped appearance of a chromosome in mitosis has become one of the iconic symbols of the life sciences (Figure 1). DNA takes its chromosome shape only during cell division, but for that time, the compaction of the genomic DNA is critical. The human genome consists of several meters of DNA: even divided between 46 chromosomes, this still leaves several centimeters to be packed into each chromosome. One might be tempted to invoke a magician's hand at work to package centimeters of DNA into a micrometer-sized chromosome. But of course, there is no magic in biology. Nor is there, yet, an answer to the question of how it is done.

Our current understanding of chromosome architecture at the molecular level is still far from complete. The only thing that becomes increasingly clear is that long-standing textbook models that show a hierarchical folding of DNA into coils and loops of increasing diameter [1], that eventually make up chromosome arms, is wrong. But if not in hierarchical coils, how is DNA folded inside a mitotic chromosome? Much of the DNA is of course incorporated into nucleosomes, but apart from that little is known about the path of DNA inside a chromosome. New genomic techniques coupled to the astounding present-day DNA sequencing power [2] and high-resolution structural analyses using a combination of modern methods [3] are beginning to give us a new sense of chromosome organization. The sense is of an irregular, flexible and self-organizing system without a predetermined folding pattern.

Thus, to appreciate the behavior of a nucleosome fiber in a chromosome we shall have to consider its biophysics. Molecular dynamics simulations will be key to understanding the implications of the biophysical constraints at a mesoscale level. Ultimately, it will be proteins and protein complexes that organize the DNA in a mitotic chromosome. Linker histones will play a role in organisms that encode them; but the density of DNA in a human chromosome is not very different from that in a bacterial nucleoid, which does not have nucleosomes. The packaging proteins that are universal are the members of the structural maintenance of chromosomes (SMC) family, which have been implicated in chromosome compaction in vertebrates and in bacteria for a long time [4-6]. How these and other proteins work on chromosomes, and indeed make a chromosome look like a chromosome, are questions that will hopefully not remain open for much longer.
